# Unconventional critical behaviour in weak ferromagnets Fe_2-x_Mn_x_CrAl (0 ≤ x < 1)

**DOI:** 10.1038/s41598-021-98377-y

**Published:** 2021-09-21

**Authors:** Kavita Yadav, Dheeraj Ranaut, K. Mukherjee

**Affiliations:** School of Basic Sciences, Indian Institute of Technology, Mandi, Himachal Pradesh 175005 India

**Keywords:** Physics, Condensed-matter physics, Materials science, Condensed-matter physics

## Abstract

Recent investigation on weak ferromagnets Fe_2-x_Mn_x_CrAl (0 ≤ x < 1) reveal the existence of a cluster glass phase (CGP) and a Griffiths-like phase (GP) below and above the ferromagnetic transition temperature (*T*_C_), respectively [(2019) *Sci. Rep.* **9** 15888]. In this work, the influence of these inhomogeneous phases on the critical behaviour (around *T*_C_) of the above-mentioned series of alloys has been investigated in detail. For the parent alloy Fe_2_CrAl, the critical exponent γ is estimated as ~ 1.34, which lies near to the ordered 3D Heisenberg class, whereas the obtained value of the critical exponent β ~ 0.273 does not belong to any universality class. With increment in Mn concentration, both exponents γ and β increase, where γ and β approach the disordered and ordered 3D Heisenberg class, respectively. The observed deviation of γ and unconventional value of δ can be ascribed to the increment of GP with Mn-concentration. The trend noted for β can be attributed to the increment in CGP regime with an increase in Mn-content. The estimated critical exponents are consistent and reliable as corroborated using the scaling law and equations of state. Our studies indicate that the critical phenomenon of Fe_2-x_Mn_x_CrAl (0 ≤ x < 1) alloys possibly belong to a separate class, which is not described within the framework of any existing universal model.

## Introduction

In the last couple of decades, the family of Heusler alloys has been extensively investigated due to novel magnetic phases like ferromagnetic, helimagnetic, Pauli paramagnetic and heavy fermionic^1–4^ exhibited by them. Interestingly, the majority of the Heusler alloys undergoes ferromagnetic transitions and tends to saturate in weak magnetic field^5^. This long-range ordering gets significantly modified by various substitutions, anti-site disorder, and variations in stoichiometry. For example, in Fe_2_V_1-x_Cr_x_Al alloys, the substitution of Cr at the V site alters the magnetic interactions between the clusters. This alternation leads to the evolution of FM ordering in this series of alloys^6^. In Fe_2_Cr_1-x_Mo_x_Al series of alloys, with Mo substitution, a significant decrement in value of the ferromagnetic transition temperature (*T*_C_) is noted^7^. In this context, analysis of critical exponent is useful to understand the role of a substitution, structural disorder, or stoichiometric variations in alteration of the FM interactions. This process has also been followed to investigate the phase transition (second order) in the Heusler alloys^7–17^. For instance, Phan et al.^8^ showed that Sn substitution at Mn site in Ni_50_Mn_50-x_Sn_x_ affects the short-range FM interactions and lead to the formation of ordered FM phase in the system. Additionally, the role of various substitutions is studied through critical exponent analysis as reported in Ni_43_Mn_46_Sn_8_Z_3_ (Z = Cr and In), Ni_47_Mn_40_Sn_13-x_Cu_x_, Ni_2.2_Mn_0.72-x_V_x_Ga_1.08_^10–12^. Furthermore, disorder also influences the critical phenomenon around FM transition^9,18–20^. In Pr_0.5_Sr_0.5-x_Ag_x_MnO_3_, it is reported that the obtained critical exponents do not belong to any conventional universal class. The increment in Ag concentration leads to an augmentation in anti-site disorder which results in the short-range interaction in the system^20^. In Ni_50_Mn_37_Sn_13_, it is observed that Gd substitution at Ni site results in the formation of long-range FM ordering^9^. Presence of disorder in transition metal-based oxide systems can lead to the formation of GP. This phase affects the long-range ordering in these systems and unconventional critical exponents are reported^21,22^. However, the effect of GP on critical exponents is still poorly understood in the Heusler alloys.

Recently, we have reported the physical properties of Fe_2-x_Mn_x_CrAl (0 ≤ x ≤ 1) Heusler alloys^23^. Our results reveal that parent alloy Fe_2_CrAl undergoes from FM to paramagnetic (PM) transition near *T*_C_ ~ 202 K. It exhibits cluster glass phase (CGP) below *T*_f1_ ~ 3.9 K and GP above T* ~ 300 K. It is observed that with Mn substitution (as x → 1) *T*_C_ shifts significantly towards lower temperature, with complete disappearance of FM behaviour in FeMnCrAl. Additionally, the alloys show CGP at low temperatures. Also, GP is found to be persistent in all alloys, with a decrement in T* with Mn concentration. Hence, it is of interest to investigate: (a) how increment of Mn content at Fe site in Fe_2_CrAl and presence of CGP influence the critical phenomena and FM interactions near *T*_C_ and (2) whether the existence of GP is always a precursor to the observed divergence in critical exponents values from the values noted in universality model. Hence, to study the aforementioned questions, in this manuscript we have investigated the critical behaviour of Fe_2-x_Mn_x_CrAl (0 ≤ x < 1) Heusler alloys in the vicinity of *T*_C_.

## Results

In order to analyse the critical phenomenon near *T*_C_, where a magnetic material undergoes a second order phase transition (SOPT) from PM state to FM state, various critical exponents are determined. In SOPT, spontaneous magnetization *M*_s_ (*T*) (below *T*_C_), initial inverse susceptibility χ^-1^_0_ (*T*) (above *T*_C_) and magnetization *M* at *T*_C_ are related to each other by the following power-law equations^24^:

1 $$M_{{S{ }}} \left( T \right) = {\text{ A}}\left( { - \varepsilon } \right)^{{\upbeta }} ,{ }\varepsilon < 0$$

2 $$\chi_{0}^{ - 1} \left( T \right) = {\text{ B}}\left( { - \varepsilon } \right)^{{\upgamma }} ,\varepsilon > 0$$

3 $$M = CH^{{\frac{1}{\delta }}} ,{ }\varepsilon = 0$$

where γ, β and δ are the value of critical exponents; A, B, and C are the constants; and ε = (*T*-*T*_C_)/*T*_C_ is the reduced temperature. From the scaling hypothesis, the relationship among *M* (*H*, *ε*), *H*, and *T* is expressed as.

4 $$M\left( {H,\varepsilon } \right) = \varepsilon^{{\upbeta }} g_{ \pm } \left( {\frac{H}{{\varepsilon^{{{\upbeta } + {\upgamma }}} }}} \right)$$

where *g*_-_(*T* < *T*_C_) and *g*_+_(*T* > *T*_C_) are regular functions^20^. The Eq. (4) can be re-written as:

5 $$m = g_{ \pm } (h)$$

where m = ε^-β^*M*(*H*,ε) and h = ε^-(β+γ*)*^*H* (m = renormalized magnetization, h = renormalized magnetic field). The above equation signifies that in case of correct choice of critical exponents and scaling relations, two separate universal curves (one below and one above *T*_C_) will be noted. This criterion is essential for validity of critical region^20^. It is also noted that the exponents lying in the asymptotic region (*ε* → 0) show universal behaviour. But the exponents often exhibit methodical trends or crossover phenomena when *T*_C_ is approached^25,26^. This appears when there is existence of disorder or couplings in the system. Due to this reason, the temperature dependent effective critical parameters (for *ε* ≠ 0) are introduced. These effective parameters show non-universal behaviour, and are given by:

6 $${\upbeta }_{\mathrm{eff}}=\frac{d[ln{M}_{S}\left(\varepsilon \right)]}{d(ln\varepsilon )},{\upgamma }_{\mathrm{eff}}=\frac{d[ln{\chi }_{0}^{-1}\left(\varepsilon \right)]}{d(ln\varepsilon )}$$

These exponents should approach universal behaviour in the asymptotic limit^25,26^.

**Figure 1:**
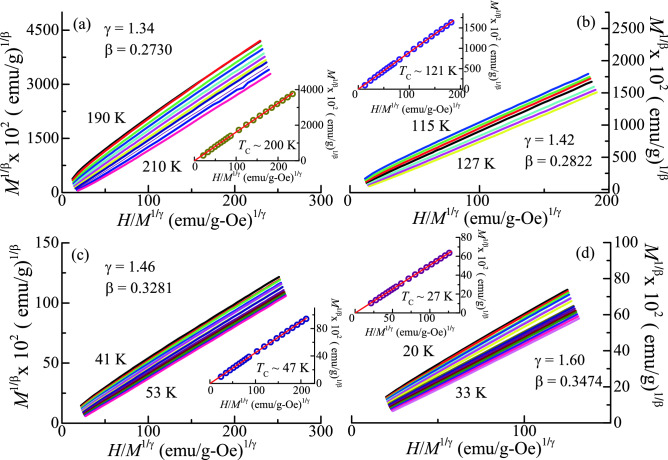
MAP (*M*^1/β^ vs *H/M*^1/γ^) of (**a**) Fe_2_CrAl, (**b**) Fe_1.75_Mn_0.25_CrAl, (**c**) Fe_1.5_Mn_0.5_CrAl, and (**d**) Fe_1.25_Mn_0.75_CrAl with estimated critical exponents (as also listed in Table 1). Insets: MAP of each alloy at *T*_C_. Red line: Linear fitting of isotherm at *T*_C_.

**Figure 2:**
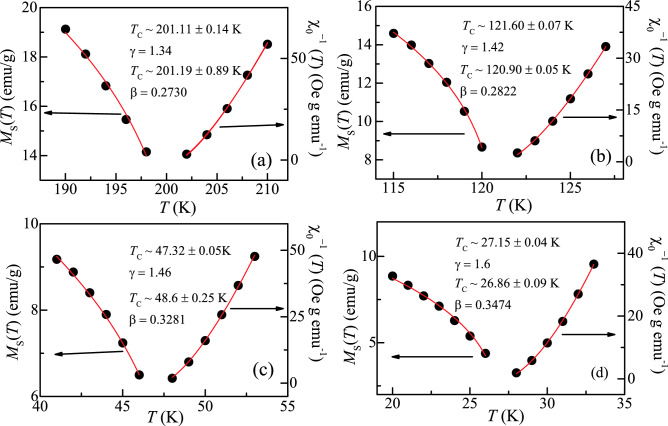
Temperature response of *M*_S_ (*T*) and χ_0_^–1^ (*T*) of (**a**) Fe_2_CrAl, (**b**) Fe_1.75_Mn_0.25_CrAl, (**c**) Fe_1.5_Mn_0.5_CrAl, and (**d**) Fe_1.25_Mn_0.75_CrAl. The critical exponents and *T*_C_ are obtained from fitting of Eqs. (1) and (2).

Conventionally, Arrott plots are used to analyse the critical region around *T*_C_. In this method, the isotherms are represented in the form of *M*^2^ vs *H*/*M,* which forms a set of parallel straight lines about *T*_C_^27^. This plot follows the mean field model (β = 0.5, γ = 1) and isotherms exhibit linear behaviour in the high field region. It also provides us the magnitude of the *M*_s_ (*T*) and χ^−1^_0_ (*T*) as an intercept on *M*^2^ and *H*/*M* axis, respectively. Arrott plot for all the alloys is plotted around *T*_C_ (shown in supplementary material Fig. S1a–d). For all the alloys, it is observed that all the curves in the plot exhibit non-linear downward curvature in the high field regime. This indicates that the critical behaviour of Fe_2-x_Mn_x_CrAl (0 ≤ x < 1) alloys cannot be described based on the mean field theory. Moreover, according to Banerjee criterion the downward curvature signifies the second order nature of the phase transition^28^. A generalized form of this analysis, known as Modified Arrott plot (MAP) involves plotting *M*^1/β^ vs *H*/*M*^1/γ^ in the critical regime. It is given by:

7 $${(\frac{H}{M})}^{\frac{1}{\upgamma }}=X\left(\frac{T-{T}_{C}}{T}\right)+Y{M}^{\frac{1}{\upbeta }}$$

where *X* and *Y* are the constants^29^. However, determination of critical exponents through this method is a non-trivial task as β and γ are two variable parameters involved in Eq. (7). This can lead to significant errors in the obtained value of exponents. Hence, for the appropriate selection of β and γ an iterative method has as suggested by Arrott et al.^29^. Therefore, to start this process, initial values of critical exponents are taken as β = 0.365 and γ = 1.386 (same as theoretical 3D Heisenberg model). The obtained values are substituted in Eq. (7) to generate a MAP. Figure 1a–d shows the MAP for the respective alloys at different temperatures. From the linear extrapolation of isotherms, the intercept on *M*^1/β^ and *H*/*M*^1/γ^ axis provide the value of (*M*_S_)^1/β^ and (*χ*_0_^–1^)^1/γ^, respectively. The *M*_S_(*T*) and *χ*_0_^–1^(*T*) values thus obtained, are utilized to fit in Eq. (1) and Eq. (2), respectively. According to these equations, the new values of β and γ can be obtained from the slope of log (*M*_S_) vs log (ε) and log(*χ*_0_^–1^) vs log(ε), respectively. It is important to note that during the straight-line fitting, *T*_C_ is adjusted in Eq. (1) and Eq. (2) such that a best fit can be obtained. Hence, to construct the new MAP, the new values of β and γ are re-used. Furthermore, to obtain the stable values of β, γ, and *T*_C_ (as listed in Table 1), this process is repeated. Using this method, for each alloy, a set of parallel isotherms has been generated. The final obtained values of *χ*_0_^–1^(*T*) and *M*_S_(*T*) are again used to estimate the values of critical exponents and *T*_C_ through scaling law. Here, for each alloy, *M*_S_(*T*) and *χ*_0_^–1^(*T*) are represented as a function of temperature as shown in Fig. 2a–d. Using these values of *M*_S_(*T*) and *χ*_0_^–1^ (*T*), the finally obtained values of critical exponents and *T*_C_ are listed in Table 1. As inferred from Table 1, the estimated values from both methods (scaling law and MAP) match well with each other.

**Table 1:** Values of the exponents β, γ, and δ as determined from the modified Arrott plot, Kouvel-Fischer plot and critical isotherm for Fe_2_CrAl, Fe_1.75_Mn_0.25_CrAl, Fe_1.5_Mn_0.5_CrAl, and Fe_1.25_Mn_0.75_CrAl.

Composition	Ref	Method	β	*T*_C_ (M_S_) K	γ	*T*_C_ (χ_0_) K	δ	*T*_C_^17^
Fe_2_CrAl	This work	MAP	0.2730	201.89 ± 0.37	1.34	201.11 ± 0.14	5.91	202 K
		KF plot	0.273 ± 0.01	201.06 ± 0.04	1.35 ± 0.02	201.01 ± 0.03	5.94 ± 0.03	
		CI	–	–	–	–	5.69 ± 0.08	
Fe_1.75_Mn_0.25_CrAl	This work	MAP	0.2822	120.90 ± 0.05	1.42	121.19 ± 0.06	6.03	120 K
		KF plot	0.282 ± 0.02	120.34 ± 0.09	1.41 ± 0.03	121.02 ± 0.02	6 ± 0.01	
		CI	–	–	–	–	5.94 ± 0.05	
Fe_1.5_Mn_0.5_CrAl	This work	MAP	0.3281	48.6 ± 0.25	1.46	47.32 ± 0.05	5.45	48 K
		KF plot	0.326 ± 0.02	46.67 ± 0.01	1.47 ± 0.05	47.41 ± 0.01	5.45 ± 0.02	
		CI	–	–	–	–	5.28 ± 0.08	
Fe_1.25_Mn_0.75_CrAl	This work	MAP	0.3474	26.86 ± 0.09	1.6	27.15 ± 0.05	5.61	27 K
		KF plot	0.345 ± 0.13	26.79 ± 0.06	1.61 ± 0.04	27.10 ± 0.04	5.60 ± 0.07	
		CI	–	–	–	–	5.56 ± 0.01	
Co_50_Cr_25_Al_25_	^13^	MAP	0.488 (3)		1.144 (4)		3.336 (5)	
		KF Plot	0.482 (13)		1.148 (16)		3.382(20)	
		CI					3.401(4)	
Co_45_Ni_5_Cr_25_Al_45_	^13^	MAP	0.513(7)		1.048(4)		3.043 (7)	
		KF plot	0.511(7)		1.028 (6)		3.012(8)	
		CI					2.976 (11)	
Co_2_TiGe	^16^	MAP	0.495		1.325		3.677	
		KF plot	0.495		1.324(4)		3.675	
		CI					3.671(1)	
Mean field theory	^32^	Theory	0.5		1		3	
3D Heisenberg model (O*)	^32^	Theory	0.365		1.386		4.80	
3D Heisenberg model (D*)	^39^	Theory	0.5		2		5	
3D Ising model	^32^	Theory	0.325		1.241		4.82	
3D XY model	^32^	Theory	0.346		1.316		4.81	

The theoretically predicted values for various universality classes are also listed for comparison.

*O ordered, *D* disordered.

**Figure 3:**
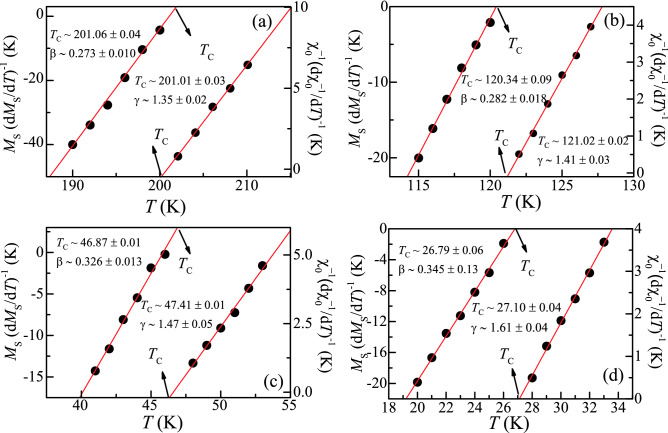
Temperature dependent *M*_S_ (d*M*_S_ / d*T*)^−1^ (left axis) and χ_0_^–1^(dχ_0_^–1^/ d*T*)^-1^ (right axis) of (**a**) Fe_2_CrAl, (**b**) Fe_1.75_Mn_0.25_CrAl, (**c**) Fe_1.5_Mn_0.5_CrAl, and (**d**) Fe_1.25_Mn_0.75_CrAl. Solid red lines denote the linear fitting.

For the exact determination of critical exponents along with *T*_C_, *M*_S_(*T*), and χ_0_^–1^ (*T*), the data is analysed using a Kouvel-Fisher (KF) plot^30^. In this method, *M*_S_ (d*M*_S_/d*T*)^-1^vs *T* and χ_0_^–1^(dχ_0_^–1^/d*T*)^-1^ vs *T* give a linear fit with slopes 1/β and 1/γ, respectively. From these plots, *T*_C_ can be easily obtained from the intercept of the fitted straight lines. KF plots for all alloys have been presented in Fig. 3a–d. The estimated exponents and *T*_C_ are listed in Table 1. The tabulated values of critical exponents and *T*_C_ estimated through KF plot and MAP matches reasonably well.

*M* (*H*, *T*_C_) vs *H* isotherms for each alloy are plotted as shown in the supplementary material Fig. S2a–d, where insets represent the same plot in log–log scale. The log *M* vs log *H* curve will show a linear variation with slope 1/δ (according to Eq. 3). The values of δ are determined from straight-line fitting. The exponent δ is estimated from the Widom-scaling relation^31^

8 $$\updelta =1+\frac{\upgamma }{\upbeta }$$

using the value of γ and β obtained from MAP studies. The obtained values of δ are 5.69 ± 0.08, 5.94 ± 0.05, 5.28 ± 0.08, and 5.56 ± 0.01 for x = 0.0, 0.25, 0.5, and 0.75 compositions. These values are very near to the value estimated from the *M* (*H*, *T*_C_) vs *H* curve. Hence, in the present study, the estimated critical exponents are accurate and do not contradict with each other. All the critical exponents estimated from different methods along with the theoretical values are given in Table 1.

**Figure 4:**
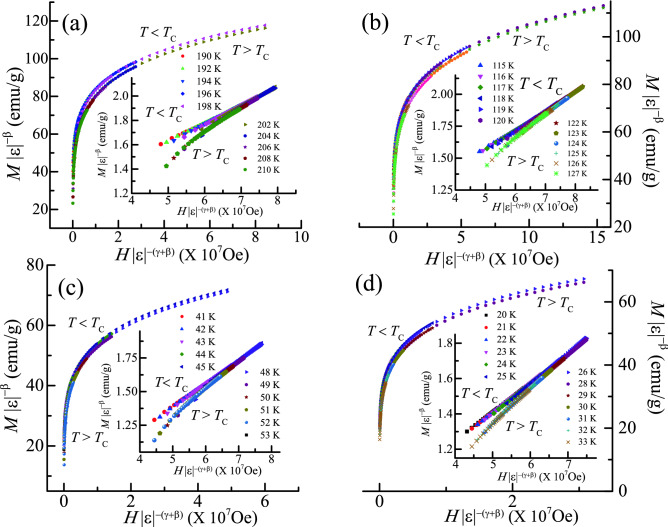
Renormalized *m* as a function of renormalized *h* (Eq. 5) with critical exponents and *T*_C_ from the Table 1 for (**a**) Fe_2_CrAl (**b**) Fe_1.75_Mn_0.25_CrAl (**c**) Fe_1.5_Mn_0.5_CrAl and (**d**) Fe_1.25_Mn_0.75_CrAl. Insets: *m* vs *h* in the log–log scale.

It is noted that the experimentally estimated critical exponents in our case do not lie within any conventional universality models. Hence, to confirm whether the obtained parameters can produce the scaling equation of state (Eq. 5), the scaled *m* as a function of scaled *h* for each alloy is shown in Fig. 4a–d. Inset of the Fig. 4a–d represents the log–log scale of the same plot. It can be clearly observed that the scaling law is satisfied in each case. All the generated isotherms diverge into two different curves: one above and one below *T*_C_. Furthermore, the consistency of the critical exponents and *T*_C_ has been re-checked using more meticulous method where *m*^2^ is represented as function of *h*/*m*^32^ as shown in the supplementary information Fig. S3a–d). For each alloy, as expected, it is observed that the data fall into two separate branches.

**Figure 5:**
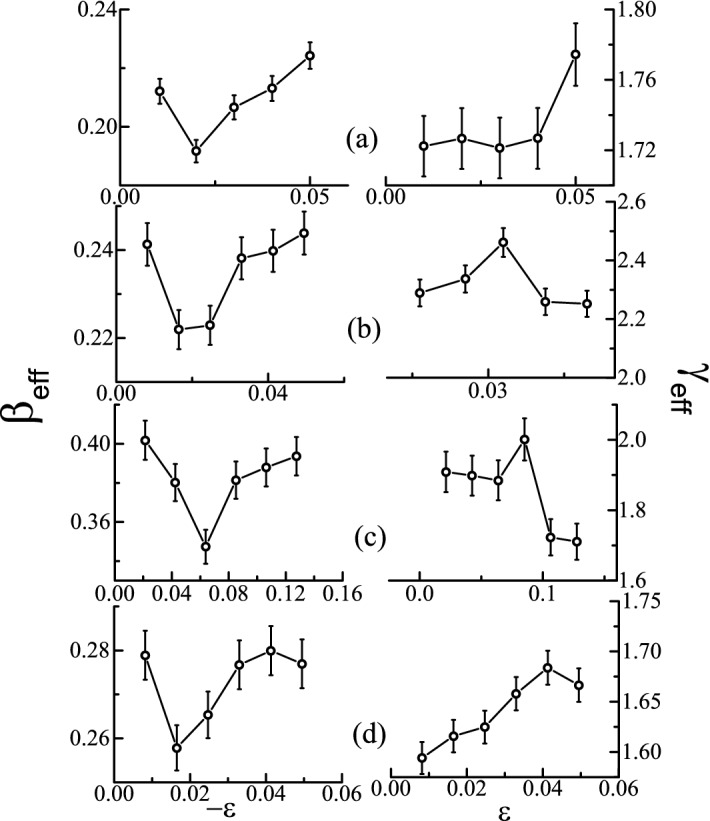
β_eff_ (left panel) and γ_eff_ (right panel) as a function of ε for ((**a**) Fe_2_CrAl, (**b**) Fe_1.75_Mn_0.25_CrAl, (**c**) Fe_1.5_Mn_0.5_CrAl, and (**d**) Fe_1.25_Mn_0.75_CrAl.

As the critical exponents obtained from various methods do not fall in any universality class, it is important to determine whether the values of γ and β match with any universal model under the asymptotic limit. Hence, we have estimated the effective critical parameters as a function of ε. It can be observed from the Fig. 5a–d that both parameters exhibit non-monotonic change with variation in ε. In case of Fe_2_CrAl, it can also be seen that β_eff_ and γ_eff_ show a slight dip (at ε = − 0.02) and a peak (at ε = 0.05) before approaching the asymptotic limit (ε → 0). A similar trend in β_eff_ and γ_eff_ for other compositions is observed. Here, the values of β_eff_ and γ_eff_ at ε_min_ do not match with any conventional universal model. Additionally, the data do not completely fall into two distinct branches, with values of β_eff_ and γ_eff_ estimated at ε_min_. This can be due to the following reasons: (i) ε_min_ does not lie in the asymptotic region and *T*_C_ must be considered more closely for asymptotic parameters or (ii) ε_min_ lies in the asymptotic regime; a similar type of disagreement of effective critical exponents (with any universal model) is also noted for other disordered materials^33,34^. In case of crystalline FM, γ_eff_(ε) shows a monotonic decrement with an increment in ε, whereas a peak is observed in amorphous FM^32^. From Fig. 5a–d, it is noted that the temperature variation of the effective critical exponents is similar to the behaviour seen in disordered FM. Thus, the above observations signify the influence of disorder on the critical exponent’s values. In Fe_2-x_Mn_x_CrAl, there is a presence of anti-site disorder between Fe and Al, which has been reported in detail in Ref.^23^. This disorder also increases with the Mn content and results in the formation of inhomogeneous magnetic phase. Hence, unconventional values of critical exponents are observed in Fe_2-x_Mn_x_CrAl .

## Discussions

In recent years, critical phenomenon is widely studied in Heusler alloys^7–17^. It has been found that the obtained values of critical exponents usually fall into a distinct universality class. For example, in Co_2_CrAl and Co_50_Ni_5_Cr_25_Al_25_ the critical exponent values (given in Table 1) are almost similar to the value as predicted by mean field model^13^. This explains the presence of long-range FM interactions in these alloys. However, the critical exponents noted for Fe_2-x_Mn_x_CrAl are unconventional and do not belong to any universality class. In our previous studies, it is reported that Fe_2_CrAl undergoes FM to PM transition around *T*_C_ ~ 202 K with the presence of GP above *T*_C_. With increment in Mn concentration, *T*_C_ decreases, and the temperature regime between FM and GP increases as shown in the phase diagram^23^. In the present study, we have noted that the values of *T*_C_ (corresponding to each alloy) obtained through various techniques matches reasonably well with the previous reported values^23^. The value of γ for the parent alloy is found to be 1.34 which is near to that reported for an ordered 3D Heisenberg model. This value increases with increasing Mn-concentration and is found to be 1.6 for Fe_1.25_Mn_0.75_CrAl. It is similar to that reported for a disordered 3D Heisenberg model. Physically, γ represents the degree of divergence of χ(*T*) at *T*_C_, smaller the value of γ, sharper will be the divergence. For the parent alloy, γ is smaller as compared to Fe_1.25_Mn_0.75_CrAl, which is in accordance to the observation of the sharp transition in the former case. Also, the larger magnitude of γ indicates the broader temperature range of PM-FM transition. The observed trend in the value of γ is consistent with the increment of temperature regime of GP. The Yang-Lee theory^21^ of phase transition predicts that the singularity of the GP can lead to unusual critical behaviour i.e., a discontinuity in the *M* (*H*) at *T* = *T*_C_. It is reflected in observed larger values of critical exponent δ. In the present case, unusual larger values of δ are noted for these alloys. This behaviour suggests that the GP affects γ and δ. As reported in Ref.^23^, because of anti-site disorder, GP arises in these alloys. Interestingly, the observed non-monotonic temperature variation of γ_eff_ also reflects the effect of disorder on the critical exponents. Here, it can be conjectured that the presence of random anti-site disorder can lead to the broader distribution of the local exchange fields due to the competition between AFM and FM exchange interactions. Similar behaviour was also noted in Fe_100-x_Pt_x_ alloy, where the value of critical exponent γ was enhanced due to the increment in the metallurgical site disorder^33^.

In Fe_2_CrAl, it is observed that there is a presence of CGP regime in the low temperature regime (below *T*_C_). This regime increases with increasing Mn-substitution. For Fe_2_CrAl, the value of β is 0.273, which does not belong to any universality class. With increment in Mn content, the value of β increases and approaches ordered 3D Heisenberg model, as found for Fe_1.25_Mn_0.75_CrAl (β = 0.347). Physically, β represents the growth of spontaneous magnetization below *T*_C,_ i.e., smaller value indicates faster growth. In the present case, the value of β is smaller for the parent alloy as compared to Fe_1.25_Mn_0.75_CrAl, implying that the growth of *M*_S_ is faster in the former alloy. With Mn substitution, the rate of growth decreases near *T*_C_, which is a consequence of increased CGP region. However, the obtained values of β (= 0.273) for Fe_2_CrAl in our case does not match well with the earlier reported value of β (= 0.42) for the same alloy^35^. This discrepancy in the obtained value of β can arise due to presence of short-range correlations (in CGP) below *T*_C_ in our case and has not been reported in the latter case.

Hence, it can be said that critical exponents for disordered ferromagnetic systems is not in accordance with any conventional universality classes. Both γ and β are affected due to the presence of GP and CGP, respectively. The unconventional behaviour of critical exponents is not unusual and has also been observed in various alloys as well as oxides. For example, a large value of β (= 0.43) is found due to phase segregation in La_1−x_Sr_x_CoO_3_ compound^36^. Similarly, Gd_80_Au_20_ exhibits unconventional exponents β = 0.44 and γ = 1.29, which arises due to spin dilution on non-magnetic ion substitution^37^. Interestingly, due to presence of GP in La_0.79_Ca_0.21_MnO_3_, larger values of γ and δ are observed^21^. Similarly, in the case of Co_2_TiGe γ and δ deviate from the 3D Heisenberg model^16^. The obtained values of these exponents (listed in Table 1) reflect the presence of sizable critical spin fluctuations. This is observed due to the existence of magnetic inhomogeneity in the alloy. Thus, our results imply that critical phenomenon in Fe_2-x_Mn_x_CrAl cannot be described based on the existing universal class models.

## Conclusion

The influence of CGP and GP on the critical exponents near the PM-FM phase transition of Fe_2-x_Mn_x_CrAl (0 ≤ x < 1) has been investigated. Each alloy exhibits a SOPT. The obtained critical exponents from different methods match well with each other. For Fe_2_CrAl, the estimated value of β is smaller than in the ordered 3D Heisenberg model, whereas γ is found to be near to this model. Along the series, both exponents β and γ show an increasing trend. For all alloys, the temperature dependences of γ_eff_ and β_eff_ resemble disordered ferromagnets, signifying the effect of anti-site disorder. This disorder induces Griffiths phase-like properties above *T*_C_, which is reflected by the unconventionally larger values of γ and δ. Additionally, the observed trend in β can be attributed to increment in CGP regime due to Mn-substitution. Our study will be helpful to comprehend the effect of inhomogeneous magnetic phases (above and below *T*_C_) on the critical behaviour of weak ferromagnetic Heusler alloys.

## Methods

The series of alloys, Fe_2-x_Mn_x_CrAl (x = 0, 0.25, 0.5, and 0.75) are the same as those reported in Ref.^23^. Structural characterization of these alloys has been already reported in Ref.^23^. From that study, we have concluded the existence of anti-site disorder in all studied alloys. Also, an increment in anti-site disorder with Mn content was also noted. Additionally, in Ref.^38^, morphological and compositional analysis of all these alloys has been carried out. It confirms the homogenous distribution of all elements in the respective alloys, which also indicates that the disorder is evenly distributed. Magnetic field (*H*) dependent magnetization (*M*) measurements have been done using Magnetic Property Measurement System (MPMS), Quantum Design, U.S.A. Rectangular shaped samples are used to obtain the *M*-*H* isotherms. The isotherms are collected in a close temperature interval (~ 1 K). Each isotherm is measured after cooling the sample from room temperature (and removal of the remanent magnetic field) to the measurement temperature. Before recording each isotherm, 10 min wait time has been given for proper stabilization of temperature.

## Supplementary Information

Supplementary Information.
