# Supplementary material for: Unconventional critical behaviour in weak ferromagnets Fe2-xMnxCrAl (0 ≤ x < 1)
Source: Sci Rep. 2021 Sep 21;11:18742. doi: 10.1038/s41598-021-98377-y (PMC8455608; doi:10.1038/s41598-021-98377-y)
Supplement: Supplementary file 1 — Supplementary Information. [file 41598_2021_98377_MOESM1_ESM.docx]

Supplementary material for

**Unconventional critical behaviour in weak ferromagnets Fe_2-x_Mn_x_CrAl (0≤x<1)**

Kavita Yadav, Dheeraj Ranaut, and K. Mukherjee

School of Basic Sciences, Indian Institute of Technology, Mandi, Himachal Pradesh-175005, India


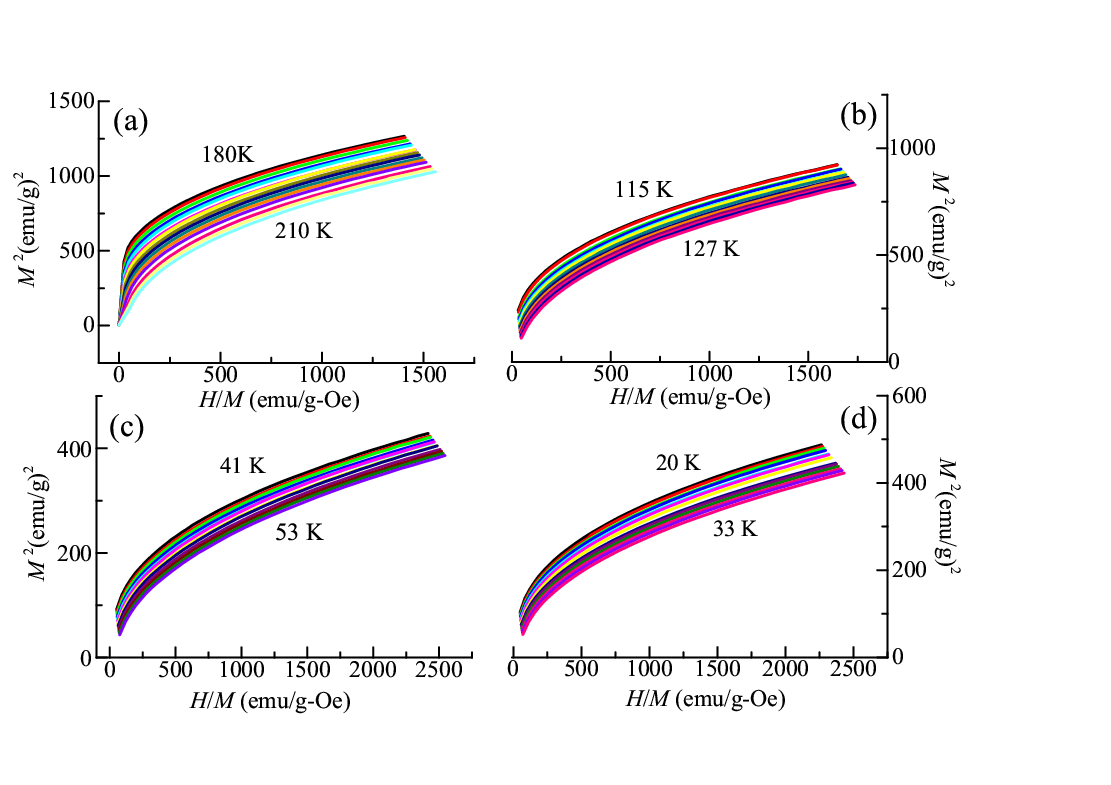


Fig. S1. Arrott plot of isotherms collected at different temperatures around *T*_C_ for (a) Fe_2_CrAl, (b) Fe_1.75_Mn_0.25_CrAl, (c) Fe_1.5_Mn_0.5_CrAl, and (d) Fe_1.25_Mn_0.75_CrAl.


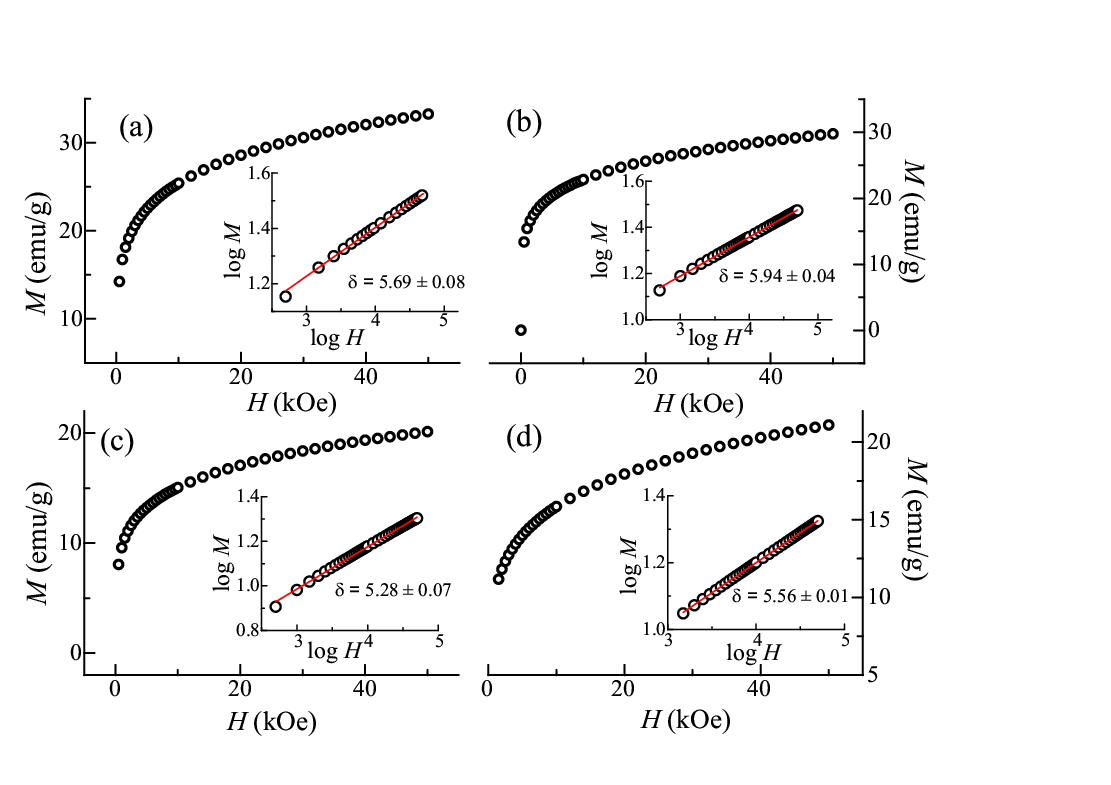


Fig. S2. *M* vs *H* plot at *T*_C_ for (a) Fe_2_CrAl, (b) Fe_1.75_Mn_0.25_CrAl, (c) Fe_1.5_Mn_0.5_CrAl, and (d) Fe_1.25_Mn_0.75_CrAl. Insets: *H* dependent *M* plot in the log-log scale. Solid red lines represent the straight-line fitting.


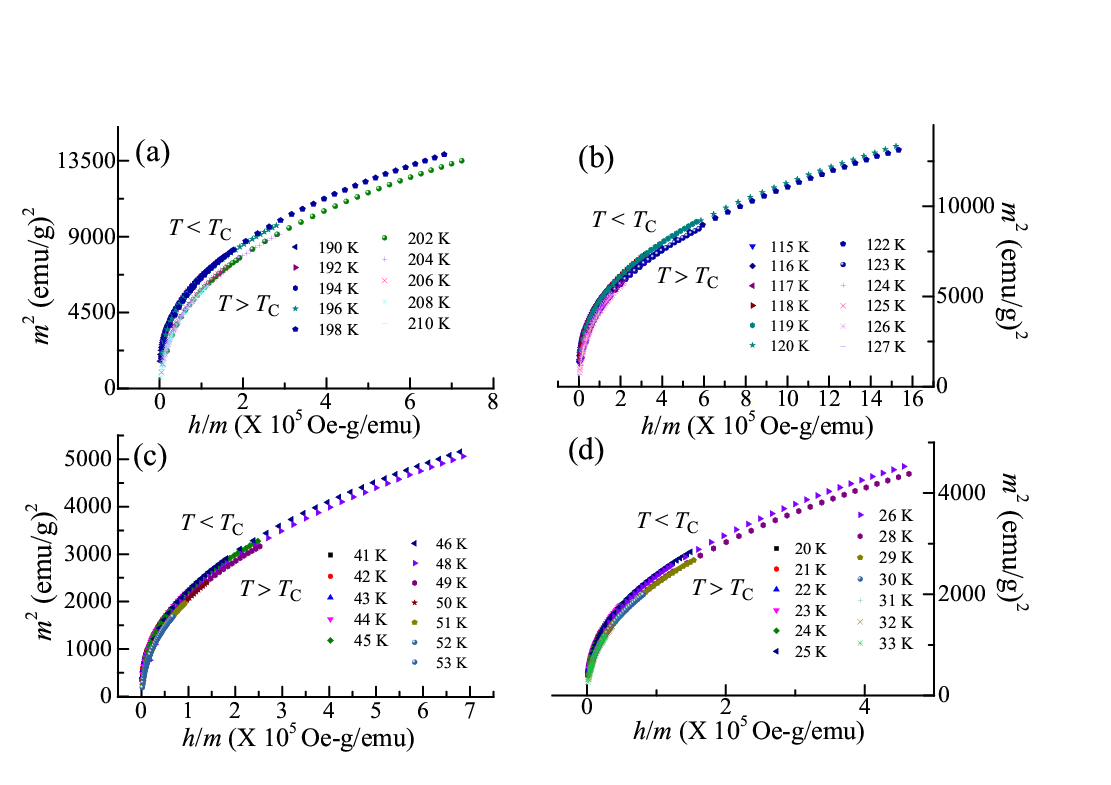


Fig. S3. *m* and *h* presented in the terms of *m*^2^ vs *h*/*m* for (a) Fe_2_CrAl, (b) Fe_1.75_Mn_0.25_CrAl, (c) Fe_1.5_Mn_0.5_CrAl, and (d) Fe_1.25_Mn_0.75_CrAl.
